# Surgical Volume in Sierra Leone: A Comparison Between Population and Facility‐Based Data Collection

**DOI:** 10.1002/wjs.70050

**Published:** 2025-08-15

**Authors:** Janine P. J. Martens, Daniel van Leerdam, Sayoh Kanneh, Isaac O. Smalle, Osman Sankoh, Alex J. van Duinen, Håkon A. Bolkan, The PRESSCO 2020 Study Group

**Affiliations:** ^1^ Hospital Group Twente (ZGT) Almelo the Netherlands; ^2^ Royal Tropical Institute Amsterdam the Netherlands; ^3^ CapaCare Masanga Sierra Leone; ^4^ Princess Christian Maternity Hospital (PCMH) Freetown Sierra Leone; ^5^ Department of Surgery University of Sierra Leone Teaching Hospital Complex (USLTHC) Connaught Hospital Freetown Sierra Leone; ^6^ University of Management and Technology (UNIMTECH) Freetown Sierra Leone; ^7^ Njala University School of Community Health Science Bo Sierra Leone; ^8^ Heidelberg Institute of Global Health (HIGH) University of Heidelberg Medical School Heidelberg Germany; ^9^ School of Public Health Faculty of Health Sciences University of the Witwatersrand Johannesburg South Africa; ^10^ Masanga Medical Research Unit Masanga Sierra Leone; ^11^ Department of Public Health and Nursing Norwegian University of Science and Technology (NTNU) Trondheim Norway; ^12^ Department of Surgery St. Olav's Hospital Trondheim University Hospital Trondheim Norway

**Keywords:** facility‐based data collection, global surgery, household survey, Sierra Leone, surgical indicators, surgical volume

## Abstract

**Introduction:**

Surgical volume obtained from health facility records is one of the six indicators proposed by the Lancet Commission on Global Surgery. An alternative approach to assess surgical volume is from household surveys, which might be more suitable for low‐income settings. The aim of this study was to describe the annual surgical volume in Sierra Leone through a population‐based approach and compare this with health facility data.

**Methods:**

This study is part of the PRESSCO 2020 (PREvalence Study on Surgical COnditions) study, a cross‐sectional countrywide descriptive study based on a randomly selected national representative sample. Data on surgeries performed annually, including type and location, were collected through interviews and compared with facility‐based data.

**Results:**

In total, 10,001 household members were included from 1854 households, who reported 152 major surgical procedures the year preceding the interview. Amounting an annual nation‐wide surgical volume of 1520 (95% CI 1300–1780) per 100,000 population. The most common procedures were hernia repairs (30.9%), caesarean sections (23.7%), and appendicectomies (13.8%). The population‐based data collection identified 4.1 times more procedures compared with 372 procedures in facility‐based study from the same year.

**Conclusion:**

The substantial difference between population‐ and facility‐based data reveals a likely underreporting in facilities and possible overreporting in the household survey. Interpretation of facility‐based surgical volume data should be carried out with caution and rigorous description of methodology is essential for proper interpretation. Independent of the method of data collection, the surgical volume in Sierra Leone is far below the recommended global benchmark.

**Trial Registration:**

ISRCTN registry under no 12353489.

## Graphical abstract

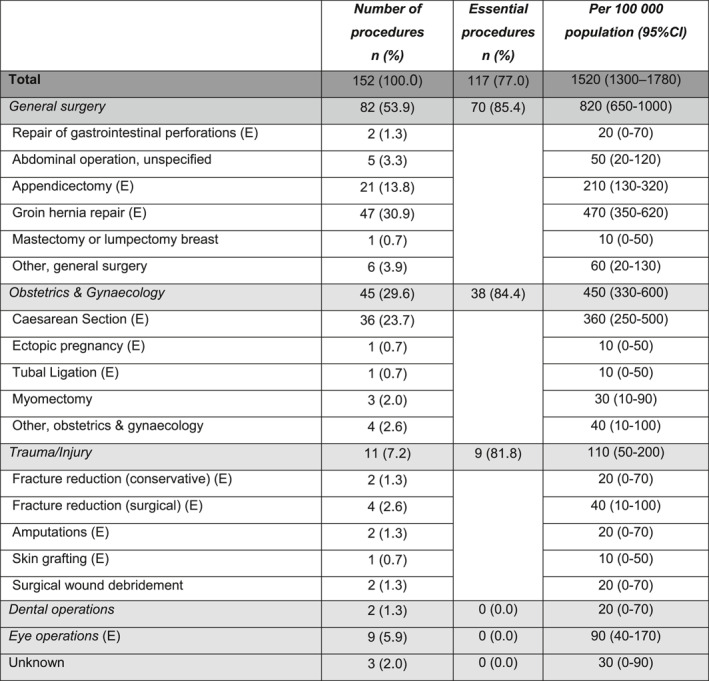

## 1 Introduction

The World Health Assembly Resolution 68/15 on “Strengthening Emergency and Essential Surgical Care and Anesthesia as a Component of Universal Health Coverage” provided a renewed focus on surgical care and urged member states to monitor its surgical care system [1]. To measure the status of a country's surgical care system and its progress toward 2030, the Lancet Commission on Global Surgery (LCoGS) proposed six surgical indicators [2]. The surgical volume indicator reports on delivery of surgical care and has been adopted in both the WHO 100 core health indicators and the World Bank's Development Indicators [3, 4]. The LCoGS recommends a minimum of 5000 surgical procedures per 100,000 population per year [2].

Although facility‐based studies have been recommended by the LCoGS as the preferred method to measure surgical volume, low‐income countries (LICs) face several challenges to track the national surgical volume indicator [2, 5]. Most LICs do not have a digital reporting systems, leading to the labor‐intensive process of manual extraction of data from paper based and often handwritten theater logbooks in each operating facility [6]. There are multiple challenges with obtaining reliable information from facilities. Poor records of facilities performing surgical procedures and challenges to obtain data from the private for‐ and nonprofit sector, which in some countries perform more than half of all surgical procedures [7]. Furthermore, there are variations in reporting practices in the operation theater logbooks leading to both overreporting and underreporting of surgical procedures [8].

An alternative approach to measure surgical volume is at the population level, which is considered the gold standard for a broad range of health indicators [9]. The aim of this study is to describe the nationwide surgical volume in Sierra Leone through a population‐based survey and to compare the findings with data obtained at the facility level [10].

## 2 Methods

### 2.1 Study Setting

Sierra Leone is a West‐African low‐income country with a population of 7.6 million, ranked 181 out of 191 on the Human Development Index [4, 11]. Life expectancy at birth is 60 years, 43.0% of the population lives below the income poverty line of 1.90 dollar per day, and more than half (55.7%) of the total health expenditures in 2020 were out‐of‐pocket spendings [12, 13].

Sierra Leone has three levels of public health care delivery. Peripheral health units (PHU's) where minor surgical procedures are performed. District hospitals serve as first referral centers for surgical care from PHUs, whereas regional and national referral hospitals at the tertiary level also provide more specialized services [13]. Finally, both private for‐ and nonprofit clinics and hospitals also offer surgical services. A recent population‐based survey on surgical conditions in Sierra Leone reported 1 in 16 inhabitants with a physical condition that warrants a visit to a surgeon and 20% of those conveyed to be disabled, unemployed, or stigmatized as result of this condition [14]. A nationwide health facility survey in Sierra Leone identified 60 locations performing major surgeries, 54 of those were classified as hospitals, of which approximately half were public facilities [10]. Based on this facility data, Sierra Leone has one of the highest recorded unmet surgical needs (92.7%) globally, reporting 372 surgical procedures per 100,000 per year in 2017 [10].

### 2.2 Study Design

This population‐based countrywide descriptive cross‐sectional household study is part of a larger initiative, the “PREvalence Study on Surgical Conditions” (PRESSCO) from 2020, where the methodology is described in detail by Van Kesteren et al. [14]. In short, sampling was done in two stages by Statistics Sierra Leone. Firstly, a total of 75 enumeration areas (EAs) were randomly selected. Secondly, within these EAs 25 households were randomly selected. In each household, the main decision‐maker was appointed by the household members themselves and became the household representative that answered questions about the other household members. Household members were defined as follows: “those that eat from the same pot and that have slept in the house last night” and were identified by the household representative and included as participants.

For each of the participants, questions were asked regarding surgical procedures that they have undergone the past 12 months preceding the interview. A household member that passed away in the last 12 months was included.

### 2.3 Outcomes

Surgical volume was defined as the number of major surgical procedures per 100,000 population [2]. Major surgical procedures were defined as the suturing, incision, excision, or manipulation of tissue that required local, regional, or general anesthesia and were conducted in an operation room [14, 15, 16]. Any manipulation of tissue either without the use of anesthesia or performed outside an operation room were considered a minor procedure, based on the definitions provided by the DCP3 essential surgical packages and Utstein consensus [17, 18].

If the household representative acknowledged that a surgical procedure was conducted for a household member in the last 12 months, first a differentiation was made between a minor and a major procedure. The household representative was then provided with a list of the most common surgical procedures, based on the DCP‐3 essential package, which was translated to Krio, the most widely spoken language in Sierra Leone. In case the surgical procedure was not identified or remembered, additional questions regarding the surgical problem and location in the body were presented to categorize the performed surgery in the broader categories of the essential surgery package.

For major surgical procedures, additional questions on the location (district) and type of facility (public or private) were asked. For private facilities, a list with all registered hospitals and clinics was obtained from the Medical and Dental Council of Sierra Leone. The list with facilities was presented to the household representative based on the district where the procedure was performed. According to the given answers, facilities were classified as “governmental,” “private registered,” or “private unregistered.” If the facility could not be identified, it was classified as “private unknown” if the household representative positively could exclude governmental facilities and “facility unknown” if not. In a separate section, all household members that passed away within the last 12 months were listed. In case the household representative acknowledged that a surgical procedure was conducted for a deceased household member in the last 12 months, the same questions regarding this procedure was asked as for living household members.

In 2018, surgical volume in Sierra Leone was assessed in a facility study where surgical logbook data for the year 2017 from 50 to 60 identified surgical facilities was included [10]. Surgical activity data were collected from all available logbooks, including operating theater logbooks, anesthesia logbooks, and maternity logbooks. The information of the logbooks was combined to acquire the most accurate and complete dataset. A more detailed description of the facility‐based data collection can be found in the article of Lindheim‐Minde et al. [10]. Results from the current household survey were compared with the results from the facility‐based study.

### 2.4 Statistical Analysis

Surgical procedures data were captured, checked, processed, analyzed, and presented as descriptive statistics using Microsoft Excel (Microsoft, Redmond, Washington, USA) and Stata 15.1 (StataCorp, College Station, Texas, USA) software. Where applicable, surgical procedures were labeled essential using the DCP‐3 classification [17] and classified as general surgery, obstetrics and gynecology, trauma/injury, dental operations, and eye operations. Annual surgical volumes per 100,000 with 95% confidence interval (CI) were calculated from the identified major surgeries divided by the surveyed population obtained from projections of the 2018 integrated household survey [19].

In accordance with the LCoGS definition on surgical volume [2], only major procedures are included for the determination of the annual surgical volume. Minor procedures are reported separately. The differences between the surgical volume obtained with the facility‐based and the household‐based approach are presented in absolute and relative differences. The unmet surgical need was calculated by subtracting the surgical volume rates form the estimated need (5000 per 100,000 populations) for surgical procedures in Sierra Leone.

### 2.5 Ethical Considerations

Ethics approval was obtained from the Norwegian Regional Committee for Medical and Health Research Ethics (REC 2019/31932), and Sierra Leone Ethics and Scientific Review Committee (SLESRC 2019/October/03). The 2017 facility study received ethical approval from the Sierra Leone Ethics and Scientific Review Committee in 2018 [10]. Only households where the household representative provided written consent were included in the study.

## 3 Results

Between October 2019 and March 2020, a total number of 10,001 household members from 1854 households were included in the study. Of these, 9647 (96.5%) were alive at the time of the interview, whereas 354 (3.5%) had passed away within the previous year.

A total of 152 major procedures, in 140 participants, were reported in the year preceding the interview, corresponding to an annual national surgical volume of 1520 (95% CI 1300–1780) per 100,000 population (Table 1). Of those, 117 (77.0%) were classified as essential procedures. The most performed procedures where hernia repair (*n* = 47, 30.9%), caesarean section (*n* = 36, 23.7%), and appendicectomy (*n* = 21, 13.8%), accounting together for more than two‐thirds (68, 4%) of all major procedures reported.

**TABLE 1:** Overview of major surgical procedures in PRESSCO 2020.

	Number of procedures *n* (%)	Essential procedures *n* (%)	Per 100,000 population (95% CI)
Total	152 (100.0)	117 (77.0)	1520 (1300–1780)
General surgery	82 (53.9)	70 (85.4)	820 (650–1000)
Repair of gastrointestinal perforations (E)	2 (1.3)		20 (0–70)
Abdominal operation, unspecified	5 (3.3)		50 (20–120)
Appendicectomy (E)	21 (13.8)		210 (130–320)
Groin hernia repair (E)	47 (30.9)		470 (350–620)
Mastectomy or lumpectomy breast	1 (0.7)		10 (0–50)
Other, general surgery	6 (3.9)		60 (20–130)
Obstetrics and gynecology	45 (29.6)	38 (84.4)	450 (330–600)
Caesarean section (E)	36 (23.7)		360 (250–500)
Ectopic pregnancy (E)	1 (0.7)		10 (0–50)
Tubal Ligation (E)	1 (0.7)		10 (0–50)
Myomectomy	3 (2.0)		30 (10–90)
Other, obstetrics, and gynecology	4 (2.6)		40 (10–100)
Trauma/injury	11 (7.2)	9 (81.8)	110 (50–200)
Fracture reduction (conservative) (E)	2 (1.3)		20 (0–70)
Fracture reduction (surgical) (E)	4 (2.6)		40 (10–100)
Amputations (E)	2 (1.3)		20 (0–70)
Skin grafting (E)	1 (0.7)		10 (0–50)
Surgical wound debridement	2 (1.3)		20 (0–70)
Dental operations	2 (1.3)	0 (0.0)	20 (0–70)
Eye operations (E)	9 (5.9)	0 (0.0)	90 (40–170)
Unknown	3 (2.0)	0 (0.0)	30 (0–90)

*Note:* (E) = Essential surgical procedure as suggested by DCP‐3.

A total of 1221 minor surgical procedures were recorded, corresponding to an incidence of 12.2% or 12,210 procedures per 100,000 population (Table 2). 81.3% of the minor procedures were essential. The most performed minor procedures were normal deliveries (*n* = 224, 20.0%), tooth extractions (240, 19.7%), and incision and drainage (*n* = 212, 17.4%).

**TABLE 2:** Overview of minor surgical procedures in PRESSCO 2020.

	Number of procedures *n* (%)	Essential procedures *n* (%)	Per 100,000 population (95% CI)
Total	1221 (100.0)	993 (81.3)	12210 (11580–12 870)
General surgery	490 (40.1)	327 (66.7)	4900 (4490–5340)
Incision and drainage of abscess (E)	212 (17.4)		2120 (1800–2400)
Male circumcision (E)	104 (8.5)		1040 (900–1300)
Urinary catheterization (E)	11 (0.9)		110 (50–200)
Dressing of wounds	135 (11.1)		1350 (1100–1600)
Punctures	28 (2.3)		280 (200–400)
Obstetrics and gynecology	244 (20.0)	244 (100.0)	2440 (2100–2800)
Normal delivery (E)	244 (2.0)		2440 (2100–2800)
Trauma/injury	182 (14.9)	182 (100.0)	1820 (1580–2100)
Suturing of laceration (E)	124 (10.2)		1240 (1000–1500)
Conservative management of fractures (E)	58 (4.8)		580 (400–700)
Dental	240 (19.7)	240 (100.0)	2400 (2100–2700)
Tooth extraction (E)	240 (19.7)		2400 (2100–2700)
Other	65 (5.3)	0 (0.0)	650 (510–830)
Unspecified	56 (4.5)		560 (400–700)
Unknown/missing	9 (0.7)		90 (100–200)

*Note:* (E) = Essential surgical procedure as suggested by DCP‐3 [16].

Close to 80% (120 of 152; 78.9%) of the major procedures were performed in the public sector against one in five (29 of 152; 19.1%) in the private sector. The household representative was able to name or recognize the name of the facility from the list for 94.4% (142 of 152) of the procedures. For three procedures (2.0%), the type of facility was unknown. Of all procedures performed in an identified private facility, less than half (13 of 29; 44.8%) were performed in facilities registered with the Medical and Dental Council. Almost one‐third (9 of 29; 31.0%) underwent their procedure in a named unregistered private facility. It was not possible to obtain information on registered or unregistered facility for about a quarter of the procedures conduced in the private setting (7 of 29; 24.1%).

Caesarean sections, appendicectomies, and hernia repairs were predominantly performed in the governmental hospitals (91.7%; 90.5%; and 70.2%, respectively). Hernia repair was the most performed procedure in the private sector (13 of 29; 44.8%). Of these, 46.2% (6 of 13; 95% CI 2–13) were performed at unregistered facilities, equaling 60 per 100,000 populations, which represents more than 4000 hernia repairs per year in Sierra Leone performed at unregistered facilities, outside of the scope of the regulating health authorities.

The population‐based data collection identified an annual surgical volume of 1520 major surgical procedures compared to 372 procedures per 100,000 population in the 2018 facility‐based study, a factor 4.1 difference (Table 3). For all procedure categories, the population‐based identified surgical volume was higher than the facility‐based. The relative difference was highest for general surgery. Caesarean sections, appendicectomies and hernia repairs combined account for 68.4% and 46.4% of the total surgical volume in the household and facility study, respectively. The number of major surgeries conducted in governmental hospitals were much higher in the population‐based survey compared to the difference observed in the private sector.

**TABLE 3:** Comparison between population and facility‐based data.

	Surgical volume (procedures per 100,000 population)		Difference	
	Facility‐based 2017 (95% CI)	Population‐based (PRESSCO 2020) 2019/2020 (95% CI)	Absolute (procedures per 100,000 population)	Relative
Total	372 (340–410)	1520 (1300–1780)	1148	4.1
By procedure category				
General surgery	143 (120–170)	820 (650–1000)	677	5.7
Obstetrics and gynecology	133 (110–160)	450 (330–600)	317	3.4
Trauma/injury (orthopedic^a^)	35 (20–50)	110 (50–200)	75	3.1
Dental operations	—	20 (0–70)	—	—
Ophthalmology	50 (40–70)	90 (40–170)	40	1.8
Other	0.4 (0–10)	—	—	—
Unknown	11 (10–20)	30 (10–90)	19	2.7
By top 3 procedures				
Caesarean section	102 (80–120)	360 (250–500)	258	3.5
Groin hernia repair	59 (50–80)	470 (350–620)	411	8.0
Appendicectomy	14 (10–20)	210 (130–320)	196	15.0
By facility				
Governmental	171 (150–200)	1200 (1000–1430)	1029	7.0
Private	201 (180–230)	290 (170–410)	89	1.4
Private registered	—	130 (70–220)	—	—
Private unregistered	—	90 (40–170)	—	—
Private unknown	—	70 (30–150)	—	—
Facility unknown	—	30 (10–90)	—	—

^a^ For the facility‐based study of 2017, only orthopedic surgeries were included.

## 4 Discussion

This is the first study comparing surgical volume from household data with facility data in a low‐income setting. The national volume of 1520 major surgical procedures performed per 100,000 population, corresponds to an unmet surgical need of 70% in Sierra Leone, far lower than the 93% previously reported by facility data [10]. Compared to other countries in the region that all have reported surgical volume from facility surveys (Liberia 462 procedures per 100,000 and Ghana 869 procedures per 100,000), Sierra Leone reported with the facility data from 2017 on the lower end with a surgical volume of 327 per 100,000 [10, 20, 21]. A global report from 2019 on the surgical volume indicator, including 72 countries though, found through facility‐based data that LICs had a mean of 328 (95% CI 231–513) procedures per 100,000 and lower‐middle‐income countries (LMICs) 2445 (95% CI 1012–4731) per 100,000 [22]. An even more recent study from 2023, focusing on LMICs only, concluded on facility‐based data that most LMICs have an average of 877 surgeries per 100,000 [5].

The remarkable four times difference between the household and the facility survey could be explained due to underreporting from the facility‐based study, overreporting within the current population‐based study, or albeit unlikely, a substantial increase in surgical volume over the 2 years separating the studies. Facility‐based studies are the recommended method by the LCoGS for the surgical volume indicator for the sake of feasibility and as it allows for comparison at facility level over time [2]. In low‐resource settings, there are several challenges that may lead to underreporting of the surgical volume reported from the facility surveys. Firstly, unregistered facilities are difficult to include in facility studies. Secondly, private facilities do not always consent to share data. Thirdly, theater logbooks in participating facilities might be incomplete, difficult to read or lost. Registration could be forgotten or even systematically avoided. Finally, charitable organizations arranging surgical camps outside the established health system is easily overlooked [23]. All those challenges contribute to an underestimation of the surgical volume when data are sampled from facilities.

The discrepancy between the household and the facility study was for this study far larger in governmental hospitals compared to private facilities (factor 7.1 vs. 1.4). Moreover, more pronounced for general surgery compared with gynecology and obstetrics (factor 5.7 vs. 3.4). In contrast to general surgery, most of the obstetrics operations are included within the country's “Free Health Care Initiative” [24]. Compared to obstetrics operations, there might be financial incentives to systematically abstain registering general surgical procedures in the hospital theater logbooks. On the other hand, overestimation in the current population‐based study is possible due to the introduction of biases. The most likely would be recall bias that might have led to overreporting of surgical procedures performed over a longer period than the last year. The final explanation of the difference between the facility survey of 2017 and the household survey of 2019/2020 is an actual increase in surgical activity within this timeframe; however, this cannot explain a fourfold increase.

The true difference between the surgical volumes will likely be a result of the combination of the three explanations outlined above. We believe the actual surgical volume is closer to 1520 per 100,000 population than the 372 per 100,000 than the facility study indicates. Household surveys are a widely accepted tool for incidence and prevalence research. The methodology for the determination of surgical volume conducted in this household survey is likely more robust compared to a facility‐based approach in a low‐resource setting such as Sierra Leone. More research in triangulating existing surgical volume data in low‐resource settings is needed to confirm this hypothesis. Nevertheless, all challenges related to establishing the surgical volume indicator from facility data, for feasibility reasons and to allow comparison over time for individual facilities, we find it sensible that facility‐based data collection remains the preferred method for the surgical volume indicator. However, this study has demonstrated a need for a review and standardization of the methodologies for facility studies of surgical volume.

The surgical volume indicator includes procedures that are performed in an operating theater using any form of anesthesia [17, 18]. However, minor surgical procedures are generally performed without (general) anesthesia and/or outside an operation theater and are therefore not included in the overall surgical volume. The incidence of minor surgical procedures in our study is 12.2%, eight times more frequent compared to major procedures. No benchmark has been set on the incidence rate of minor surgical procedures. Also, to our knowledge, no data on nationwide level for low‐income countries specifically on minor procedures has been described yet. The preventive value of certain minor procedures, such as tooth extractions and incision and drainage, has been widely recognized [25, 26]. A timely, decent quality, minor procedure could prevent less severe surgical condition escalating to more severe conditions. Because of their feasibility to organize at primary level, promoting these can be considered a cost‐effective intervention. Monitoring of the minor surgical procedures is the first step and can provide useful information for surgical system planning.

Almost 6% of the surgical volume was performed in private facilities that are not registered with the Sierra Leone Medical and Dental Council. A further 5% of the major procedures was performed in unknown private facilities. This means that up to 10 percent of the surgical volume in Sierra Leone or 10,000 surgeries annually are performed in a facility with limited possibilities for oversight by regulation health authorities. This has serious implications on the quality of the care offered.

### 4.1 Strengths and Limitations

A strength of this study is that interviews and examinations were conducted by local trained healthcare personnel together with experienced staff from Statistics Sierra Leone, familiar with the Demographic and Health Survey (DHS). Another strength was a rigorous system for data checking and validations, carried out by a data quality team consisting of field supervisors that cross‐checked the data upon completion of the household and core study group members cross‐checking at the EA level. The expansion of Sierra Leone's telephone network during the interim period has facilitated the ease of transmission and verification of study data.

This study has important limitations to consider. First, biases could have been introduced because of self‐reporting. The most important is the recall bias that might have led to overestimation or underestimation of surgical procedures performed the last year, particular for the minor surgical procedures. Major surgery, on the other hand, is a major incident with considerable financial implications for the household, it is a sporadic event and combined with the relatively short recall period of 1 year, we would argue that this minimized the bias [27, 28, 29]. Social desirability bias could occur as the questionnaire might contain sensitive questions (e.g., disclosure of informal procedure location). To address this, local interviewers were specifically trained to be sensitive and to avoid prejudging openly. Observer bias, introduced by the interviewer altering responses during input, was also attempted to mitigate by thorough selection and training of the interviewing personnel. Secondly, although the survey instrument was based on the validated SOSAS questionnaire, the methodology for this particular study on previous surgical operations was designed by this group and is not externally validated. Thirdly, we adhered to the experiences of Statistics Sierra Leone and the national DHS‐protocol by allowing the interviews to be held in the languages preferred by the one being interviewed. We recognize that the use of the English language for the questionaire and not translating it to all major local tribal languages was another limitation. This was mitigated by translating all major surgical procedures to Krio by local surgical providers for a better understanding.

## 5 Conclusion

This household study demonstrated a four times higher national surgical volume than previously reported in Sierra Leone through facility‐based data collection. Lack of triangulation of surgical volume data from comparable LICs indicate that the surgical volume indicators from facility‐based data in low resource setting should be interpreted with care. We recommend integrating the surgical volume indicator as a standardized and validated part of the DHS to be able to secure quality and routine population‐based data collection that can be compared between countries and time. At this moment though, we do not challenge a facility approach for reporting surgical volume as the preferred method but call for a review and standardization of the methodologies for facility studies of surgical volume. Regarding this study, Sierra Leone, with a surgical volume of 1520 surgeries per 100,000 population, did not reach the benchmark set by the LCoGS. Also, there is a considerable amount of major surgical procedures being performed every year in unregistered health facilities, outside of the scope of regulation health authorities. Additional research is necessary to obtain better insight of the full volume and the quality of these practices in LICs.

## Author Contributions

**Janine P.J. Martens:** conceptualization, data curation, formal analysis, investigation, methodology, project administration, writing – original draft, writing – review and editing. **Daniel van Leerdam:** conceptualization, data curation, formal analysis, investigation, methodology, project administration, writing – original draft, writing – review and editing. **Sayoh Kanneh:** data curation, investigation, methodology, writing – original draft, writing – review and editing. **Isaac O. Smalle:** conceptualization, methodology, supervision, supervision, writing – original draft, writing – original draft, writing – review and editing, writing – review and editing. **Osman Sankoh:** conceptualization, data curation, formal analysis, methodology, supervision, writing – review and editing. **Alex J. van Duinen:** conceptualization, formal analysis, investigation, methodology, supervision, writing – original draft, writing – review and editing. **Hakon A. Bolkan:** conceptualization, data curation, formal analysis, funding acquisition, investigation, methodology, project administration, supervision, validation, writing – original draft, writing – review and editing.

## Consent

Informed consent was obtained from all individual participants included in the study.

## Conflicts of Interest

The authors declare no conflicts of interest.
